# Roborovski hamster *(Phodopus roborovskii)* strain SH101 as a systemic infection model of SARS-CoV-2

**DOI:** 10.1080/21505594.2021.1972201

**Published:** 2021-09-14

**Authors:** Chongkai Zhai, Mingda Wang, Hea-Jong Chung, Mehedi Hassan, Seungkoo Lee, Hyeon-Jin Kim, Seong-Tshool Hong

**Affiliations:** a Department of Biomedical Sciences and Institute for Medical Science, Jeonbuk National University Medical School, Jeonju, South Korea; b Gwangju Center, Korea Basic Science Institute, Gwangju, South Korea; c Department of Anatomic Pathology, School of Medicine, Kangwon National University, Kangwon National University Hospital, Chuncheon, South Korea; d JINIS BDRD Institute, JINIS Inc, Bongdong, South Korea

**Keywords:** SARS-CoV-2, roborovski hamster, *Phodopus roborovskii* SH101, COVID-19 animal model

## Abstract

Severe acute respiratory syndrome CoV-2 (SARS-CoV-2) is currently causing a worldwide threat with its unusually high transmission rates and rapid evolution into diverse strains. Unlike typical respiratory viruses, SARS-CoV-2 frequently causes systemic infection by breaking the boundaries of the respiratory systems. The development of animal models recapitulating the clinical manifestations of COVID-19 is of utmost importance not only for the development of vaccines and antivirals but also for understanding the pathogenesis. However, there has not been developed an animal model for systemic infection of SARS-CoV-2 representing most aspects of the clinical manifestations of COVID-19 with systemic symptoms. Here we report that a Roborovski hamster strain SH101, a laboratory inbred hamster strain of *P. roborovskii*, displayed most symptoms of systemic infection upon SARS-CoV-2 infection as in the case of the human counterpart, unlike current COVID-19 animal models. Roborovski hamster strain SH101 post-infection of SARS-CoV-2 represented most clinical symptoms of COVID-19 such as snuffling, labored breathing, dyspnea, cough, hunched posture, progressive weight loss, ruffled fur, and high fever following shaking chills. Histological examinations also revealed initial right-predominated pneumonia as well as slight organ damages in the brain and liver, manifesting systemic COVID-19 cases. Considering the merit of a small animal as well as its clinical manifestations of SARS-CoV-2 infection in human, this hamster model seems to provide an ideal tool to investigate COVID-19.

## Introduction

The emergence of COVID-19 upon the infection of SARS-CoV-2 has been posing a mounting threat to the world since 2019. In COVID-19, the infection of SARS-CoV-2 in the respiratory system causes fever, shaking chills, headache, and fatigue followed by respiratory symptoms such as cough, sneeze, sore throat, chest pain, and atypical pneumonia [1,2]. The clinical features of COVID-19, however, are not limited to the consequences of respiratory infection [3]. Unlike a typical respiratory infection, SARS-CoV-2 infects organs other than the respiratory system from the beginning or subsequently to respiratory infection, leading to diarrhea, loss of sense of smell or taste, neuroinflammation manifested as encephalitis, meningitis, acute cerebrovascular disease, and Guillain Barré Syndrome (GBS), and multisystem inflammatory syndrome [4–6]. Moreover, atypical pneumonia by SARS-CoV-2 is observed with heavy predominance in the right lung [7]. The right-over-left predominance in pneumonia is one of the hallmarks of COVID-19 [8,9]. As the consequences of these systemic infections, patients who recovered from severe or even a mild form of COVID-19 frequently suffer from fatigue, heart palpitations, changes in lung function, muscle weakness, memory loss, concentration, brain fog, depression, anxiety [10–12].

The mutation rate in RNA virus such as SARS-CoV-2 is dramatically high, up to a thousand times higher than that of DNA virus, contributing to rapid evolution into its variants. Considering the nature of an RNA genome and the number of confirmed cases of the outbreak, the emergence of new strains from furtively circulating SARS-CoV-2 seems to be inevitable even after the establishment of herd immunity through vaccination. In fact, the world is already observing the emergence of multiple variants of SARS-CoV-2. Given the number of confirmed cases, an appropriate surrogate for human COVID-19 is needed to overcome the imminent threats of COVID-19 from a future emergence of variant strains. Moreover, animal models could help shed light on the important aspects of human COVID-19 in ways that are not easily addressed or feasible in humans, such as how SARS-CoV-2 causes systemic infection.

After the outbreak of COVID-19, various animal models for COVID-19 have been developed. The current animal models such as hACE2 transgenic mice, hamsters, ferrets, fruit bats, guinea pigs, African green monkey, Rhesus macaques, and Cynomolgus macaques supported SARS-CoV-2 replication and displayed varying degrees of illness when the virus was delivered into the respiratory tract of these animals [13–17]. However, the infections of SARS-CoV-2 in these animals were mainly limited to the respiratory systems. Since these animal models mostly recapitulated the infections of the respiratory system, each of these animals has a limited utility in the study of COVID-19 [18–22]. Therefore, the development of an animal model recapitulating systemic infection, including COVID-19-specific facet such as right-predominated pneumonia, would be needed utmostly to overcome the imminent threat COVID-19 poses.

In this work, we identified a laboratory-inbred strain of Roborovski hamster (*P. roborovskii)* strain SH101, representing the systemic infection of human COVID-19. In addition to systemic infection, the infection of Roborovski hamster SH101 with SARS-CoV-2 captured almost all clinical symptoms represented by human COVID-19 including high fever, shaking chills, serious right-predominated pneumonia, and the viral distribution pattern as well as evident respiratory and behavioral symptoms.

## Methods

### Ethics approval

All procedures involving the mice and hamsters were accredited with the approval of the Institutional Animal Care and Use Committee (IACUC), in compliance with the guidelines of the Ethics Committee of Jeonbuk National University Laboratory Animal Center Guidelines on the Care and Use of Animals for Scientific Purposes. All animal experiments in this study were in accordance with the ARRIVE guidelines and checklist. All the animals used in this research were handled in a manner consistent with CDC/ABSA/WHO guidelines for the prevention of human infection with the SARS-CoV-2 virus.

### Viruses and cells

The SARS-CoV-2 strain HB-01 was obtained from the National Culture Collection for Pathogens (NCCP) of the Korea Disease Control and Prevention Agency (KDCA). The complete genome for this SARS-CoV-2 has been submitted to GISAID (identifier: BetaCoV/Wuhan/IVDC-HB-01/2020|EPI_ISL_402119) and deposited in the China National Microbiological Data Center (accession number NMDC10013001 and genome accession number MDC60013002-01). Preparation of seed SARS-CoV-2 stocks and isolation of the virus were performed in Vero cells, which were maintained in Dulbecco’s modified Eagle’s medium (DMEM) supplemented with 10% fetal bovine serum (FBS), 100 IU/mL penicillin, and 100 μg/mL streptomycin, and incubated at 37°C, 5% CO_2_.

### Animal experiments

Six-week-old hACE2 transgenic mice (K18-hACE2 strain) with human angiotensin I-converting enzyme (peptidyl-dipeptidase A) 2 (hACE2) gene under the human cytokeratin 18 (K18) promoter [23] on chromosome 2 (99,209,508–99,220,724) and Syrian golden hamsters (*Mesocricetus auratus*) were purchased from Alpha biochemicals Co (http://alphabiochemicals.com). Roborovski hamster (*P. roborovskii)* strain SH101 was a laboratory inbred hamster strain maintained in Jinis Biopharmaceuticals Inc. (Wanju, Jeonbuk, Republic of Korea). We deposited *P. roborovskii* SH101 for distribution in Alpha biochemicals Co (http://alphabiochemicals.com).

For animal experiments, three animals of each animal species were housed in one cage and were provided with food (10% kcal as fat intake; D12450B; Research Diets Inc.) and water ad libitum. A 12-hour light-dark cycle was maintained (lights on at 8:30 PM daily) in a room of the ABL3 lab of Jeonbuk National University with controlled temperature (22°C ± 1°C) and humidity (55% ± 5%) for the animals. After 2 weeks of acclimation in the ABL3 lab, 50 μL of SARS-CoV-2 solution containing 10^5^ TCID_50_ or mock (DMEM) was inoculated intranasal by using a pipette. The food consumption of each mouse group was monitored daily. For blood profiling and behavioral tests, animals were randomly assigned to each treatment group. Particularly, we declare that blinding was employed during animal allocation and data collection.

### Body temperature measurement

The body temperatures of the animals were measured by a high-precision thermal photographic method [24,25]. Each of the whole animal bodies was photographed by FLIR thermal imaging camera, and the thermal images were analyzed by DirA Program (FLIR Tools) to measure the body temperature. The highest temperature spot on the chest, close to the site of the lung, was allocated in each mouse to determine the body temperature. All data are presented as the mean ± standard deviation and were compared using paired Student’s t-tests.

### D-dimer and fibrin degradation products (FDPs) analysis

The hypercoagulable state of the SARS-CoV-2-infected animals was analyzed daily by D-dimer and FDPs assays during the experimental period. Whole blood was collected in a 1.5 mL Eppendorf micro-centrifuged tube by cardiac puncture. Blood serum was separated by centrifugation at 2,500 × g for 10 min and stored at 25°C until used. The D-dimer and FDPs concentrations of serum samples were determined by the double antibody sandwich method using mouse D-dimer and mouse FDPs ELISA kits (Sunlong Biotech, Hangzhou, Zhejiang, China) as described previously [26]. Briefly, 20 ~ 23 times diluted sera with the dilution buffer were used to quantitate D-dimer or FDPs in a micro-ELISA strip plate pre-coated with mouse anti-D-dimer or anti-FDPs monoclonal antibody. The concentrations of D-dimer and FDPs were calculated from the OD values of each sample using a standard curve.

### Preparation of total RNAs from the primary organs

After the euthanization of the experimental groups, each mouse was dissected to isolate primary organs; lung, brain, liver, pancreas, kidney, stomach, trachea, spleen, small intestine, heart, and colon. Tissue homogenates (100 mg/mL) were prepared by homogenizing perfused organs using an electric homogenizer for 2 min 30 s in DMEM. The homogenates were centrifuged at 3,000 × rpm for 10 min at 4°C. The supernatants were collected and stored at −80°C until virus quantifications.

### Quantitation of SARS-CoV-2 titers

Since Real-time qPCR (RT-qPCR) is widely used as an alternative for virus counting [27], we used a RT-qPCR method for quantification of SARS-CoV-2. Total RNA was extracted from the supernatants of the organ homogenates using the RNeasy Mini Kit (QIAGEN, Hilden, Germany), and reverse transcription was performed using the PrimerScript RT Reagent Kit (TaKaRa, Japan) following the manufacturers’ instructions. RT–qPCR reactions were performed using the PowerUp SYBG Green Master Mix Kit (Applied Biosystems, Waltham, MA, USA), in which samples were processed in duplicate using the following cycling protocol: 50°C for 2 min, 95°C for 2 min, followed by 40 cycles at 95°C for 15 s and 60°C for 30 s, and then 95°C for 15 s, 60°C for 1 min, 95°C for 45 s. The primer sequences used for RT–qPCR is targeted against the envelope (E) gene of SARS-CoV-2 and are as follows: forward: 5′-GCCTCTTCTCGTTCCTCATCAC-3′, reverse: 5′- AGCAGCATCACCGCCATTG −3′. The PCR products were verified by sequencing using the dideoxy method on an ABI 3730 DNA sequencer (Applied Biosystems, Waltham, MA, USA). The obtained sequencing reads were compared with the reads from the NCBI database. The SYBR green real-time PCR standard curve was generated by serial ten-fold dilutions of recombinant plasmid with a known copy number (from 7 ×10^7^ to 7 × 10^1^ copies per μL). These dilutions were tested and used as quantification standards to construct the standard curve by plotting the plasmid copy number against the corresponding threshold cycle values (Ct). Results were expressed as log_10_-transformed numbers of genome equivalent copies per ml of sample. The Ct values of each sample were used to quantitate the virus titers by using the standard curve.

### H&E staining and immunohistochemistry

About halves of the primary organs (lung, brain, liver, pancreas, kidney, stomach, trachea, spleen, small intestine, heart, and colon) were isolated from 3 adult postmortem *P. roborovskii* SH101 at 2 dpi (euthanized hamsters) and 4 dpi (hamster carcasses at terminal stage), respectively. The same primary organs were isolated for histological examination from 2-month-old *P. roborovskii* SH101 carcasses at 4 dpi, euthanized hACE2 transgenic mice at 7 dpi, and euthanized Syrian golden hamster at 7 dpi. Immediately after isolation, all organs were fixed using 10% neutral-buffered formalin, embedded in paraffin, and sectioned to be placed on glass microscopic slides (5 μm). After the removal of paraffin from the sections on the microscopic slides by hot water, the slides were air-dried and baked overnight at 65°C. The organ sections were stained with Hematoxyline and Eosin (H&E) with standard staining procedure [28,29]. The stained tissue images were observed in Apero ScanScope FL (Leica Biosystems, Germany).

For immunohistochemistry (IHC), The embedded tissues were routinely cut into 4 μm thick sections. Deparaffinized, rehydrated tissue sections were incubated in antigen retrieval buffer for 15 min at 97°C and endogenous peroxidase was quenched using 3% H_2_O_2_ in methanol for 10 min. Nonspecific protein binding was blocked using 5% normal goat serum and then the sections were incubated with SARS-CoV-2 nucleocapsid rabbit monoclonal antibodies (40143-R001, Sino Biological) at 4°C overnight, followed by horseradish peroxidase (HRP)-labeled goat anti-rabbit IgG secondary antibody (32460, Invitrogen). The sections were developed with peroxidase substrate kit DAB (SK-4100; Vector), and counterstained with hematoxylin, dehydrated in ethanol and xylene, and glass coverslips mounted using Dibutyl Phthalate Xylene.

### Statistical analysis

All three animal models were analyzed with the same analysis method as described here. The statistical significance of test samples from SARS-CoV-2-infected or mock-treated animal groups was assessed by a one-way ANOVA multiple comparisons test. For the body temperature measurement, data are presented as the mean ± standard deviation and were compared using paired Student’s t-tests. In addition, statistical analyses were performed using GraphPad Prism 5 (GraphPad Software, La Jolla, CA, USA). A *p*-value less than 0.05 was considered statistically significant.

## Results

### A hamster strain recapitulating COVID-19 after SARS-CoV-2 infection was identified

**Figure 1.**
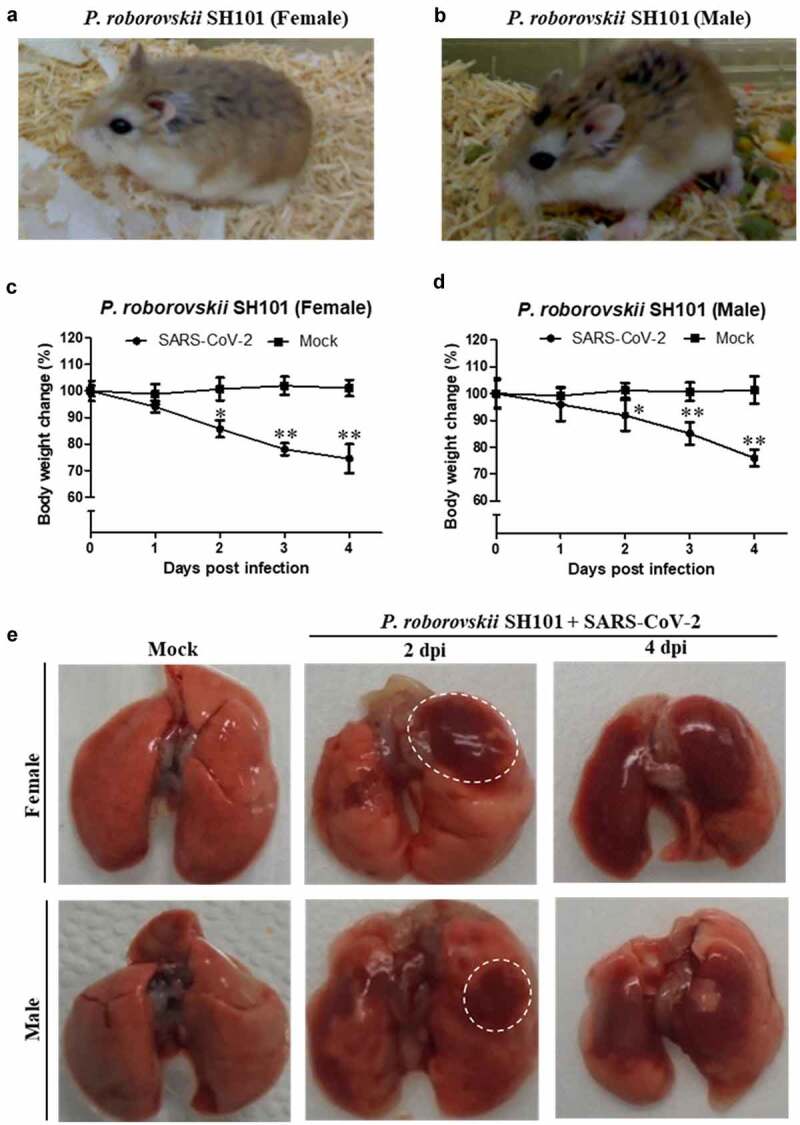
Identification of a small animal model for SARS-CoV-2 infection representing most clinical features of COVID-19. (a, b) The photographic image of adult female (a) and male (b) Roborovski hamster SH101, a laboratory inbred strain. (c, d) The body weight changes for 2-month-old female (c) and male (d) Roborovski SH101 post-infection of SARS-CoV-2. The body weights were measured daily for 5 days (up to 4 dpi) (n = 6). Data are presented as mean ± SD. The statistical significances are marked on the graphs as * *P* < 0.05 and ** *P* < 0.01. (e) The photographic images of the dissected lungs of the SARS-CoV-2-infected hamster with right-predominant pneumonia indicated as white dotted circles

To develop an ideal animal model of COVID-19, we screened several hundred strains of mice, rats, guinea pigs, and hamsters based on mortality, and identified a Roborovski hamster (*Phodopus roborovskii)* SH101, which was highly sensitive to SARS-CoV-2 infection. Roborovski hamster strain SH101 (abbreviated as SH101) is a laboratory-inbred hamster strain with prominent white patches above eyes and at the base of ears (Figure 1(a,b)). The average adult body weights of SH101 were about 21 g for males and 20 g for females, respectively.

As in the case of COVID-19, respiratory symptoms were immediately noticed in the SH101 hamsters infected with SARS-CoV-2 (Video S1~ S4, also refer to the summary in Supplemental Table 1). The hamsters showed clear signs of respiratory symptoms such as snuffling, dyspnea, cough, labored breathing, ruffled fur, and sneeze. Along with the respiratory symptoms, the very active behavior otherwise typical for the hamsters was dramatically reduced while hunched posture was observed starting from 1-day post-infection (dpi). Other than these clinical manifestations associated with severe respiratory and systemic infection, some SH101 hamsters showed shaking chills (Video S3) after 2 dpi which resembled the shaking chills of human patients of COVID-19. Also, the progressive and significant weight loss had been observed from 2 to 4 dpi (Figure 1(c,d)). All individuals were terminally ill, and the mortality rate of the SH101 hamsters was 83% by 4 dpi.

More interestingly, a unique uneven distribution of pneumonia was noticed immediately by gross examination of the lung specimens (Figure 1(e)). The dissected lungs at 2 dpi of the infected SH101 hamsters showed right-predominated pneumonia while bilateral pneumonia could be observed at 4 dpi. It was remarkably interesting to note the right-predominated pneumonia of the hamster because one of the most peculiar clinical manifestations of COVID-19 is the right-over-left predominated pneumonia [30–32].

### Roborovski SH101 infected with SARS-CoV-2 progressed similarly with human COVID-19

**Figure 2.**
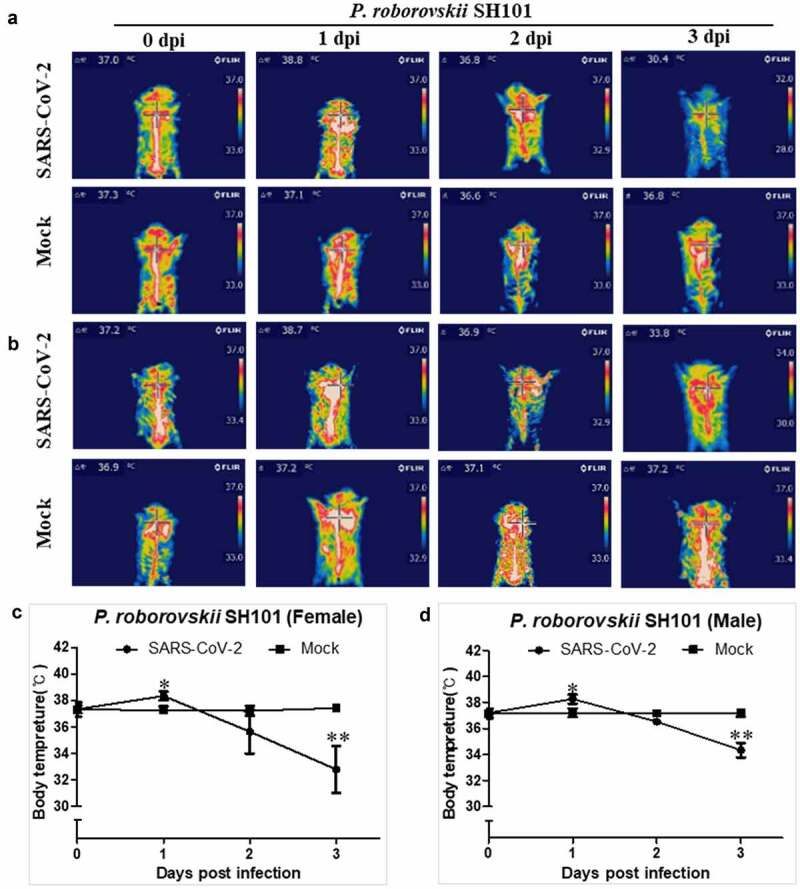
The body temperature changes of Roborovski SH101 hamster infected with SARS-CoV-2. (a, b) The representative infrared thermographic images of the 2-month-old female (a) and male (b) *P. roborovskii* SH101 for 0, 1, 2, 3 days post-infection (dpi) of SARS-CoV-2. (c, d) The body surface temperatures on the chest, as close as possible to the lung, of the 2-month-old female (c) and male (d) *P. roborovskii* SH101 hamster infected with SARS-CoV-2 (n = 6). The body temperatures were measured by selecting the highest temperature spot on the thermal images and are presented as mean ± SD. The statistical significances are marked on the graphs as * *P* < 0.05 and ** *P* < 0.01

Although the induction of fever in COVID-19 is an essential hallmark of SARS-CoV-2 infection, none of the current animal models, including primates, showed an induced fever by SARS-CoV-2 infection. In this study, the body temperature measurement by an infrared thermographic method revealed the induction of fever (above 37.5℃) in SH101 hamsters immediately after infection of SARS-CoV-2 (Figure 2). The elevated body temperatures of the infected SH101 hamsters were sharply dropped as the infection progressed to the terminal stage. Being one of the cardinal features in COVID-19 [33,34], the fever induction after SARS-CoV-2 infection seems to indicate that the SH101 hamsters simulate COVID-19 symptoms most closely among animal models of COVID-19.

**Figure 3.**
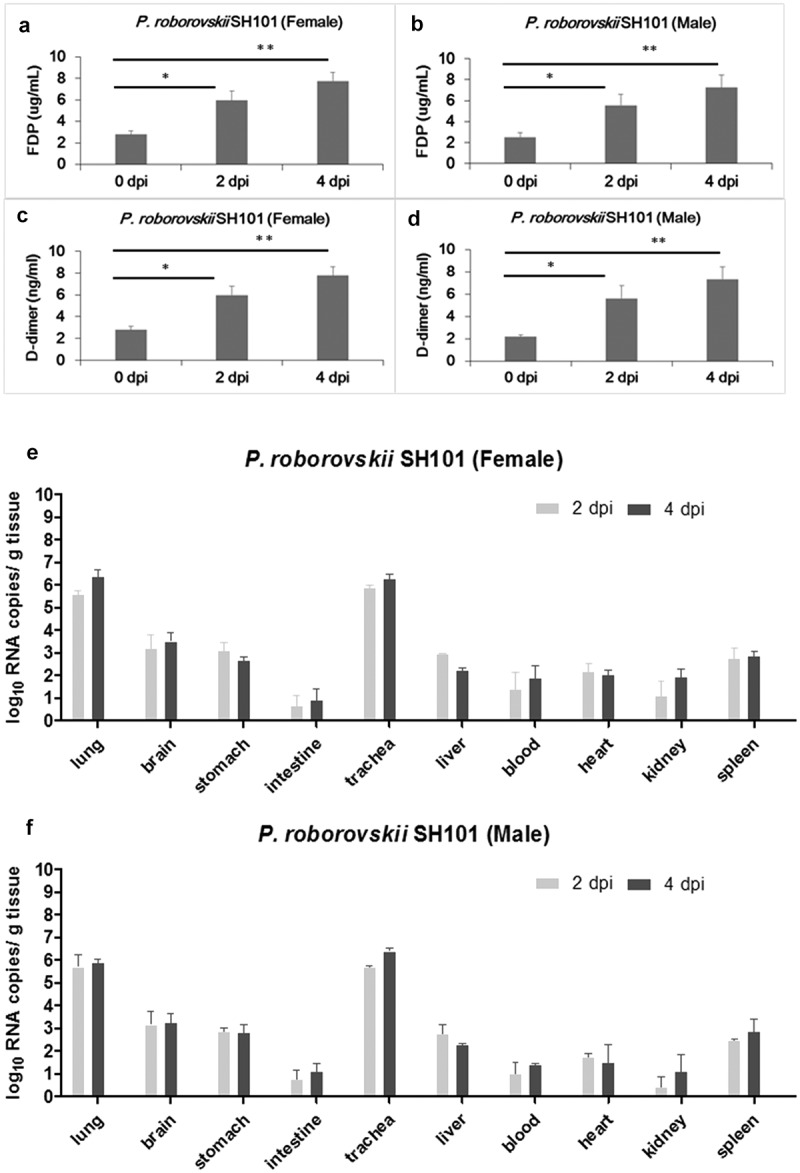
Thrombosis and viral replication in Roborovski SH101 hamster infected with SARS-CoV-2. (a, b) The levels of fibrin degradation products (FDP) in the plasma of the female (a) and male (b) *P. roborovskii* SH101 hamsters at 2 and 4 dpi of SARS-CoV-2 (n = 3). (c, d) The levels of D-dimer in the plasma of the female (c) and male (d) *P. roborovskii* SH101 hamsters at 2 and 4 dpi of SARS-CoV-2 (n = 3). (e, f) The viral RNA levels in the lung, brain, stomach, intestine, trachea, liver, blood, heart, kidney, and spleen of the female (e) and male (f) *P. roborovskii* SH101 hamsters measured by RT-qPCR at 2 and 4 dpi of SARS-CoV-2. Data are presented as mean ± SD (n = 3). The statistical significances are marked on the graphs as * *P* < 0.05 and ** *P* < 0.01

Another unusual feature of human COVID-19 is the widespread thrombosis of small and large vessels, contributing to morbidity and mortality [35–37]. It was also known that fibrinolysis was elevated in human COVID-19 due to systemic multiorgan thrombosis by SARS-CoV-2 infection [38,39]. The levels of fibrin degradation products, D-dimer and FDP, were elevated in the plasmas of the SH101 hamsters infected with SARS-CoV-2 (Figure 3a-D)), suggesting the occurrence of systemic infection in the hamsters as in the case of human COVID-19.

We next quantitated SARS-CoV-2 in the SH101 hamsters by quantitative RT-PCR after reverse transcription (RT–qPCR). The primary organs were collected from 3 randomly assigned individuals in each group at 2 and 4 dpi for analysis. High levels of viral RNA were detected in the homogenates of the lung and trachea, whereas lower levels were detected in the brain, stomach, intestine, liver, blood, heart, kidney, and spleen (Figure 3(e,f)). The detection of the viral RNAs in all organs from the SH101 hamsters confirmed serious systemic infection caused by SARS-CoV-2.

### The histological examinations of P. roborovskii SH101 Infected with SARS-CoV-2 revealed severe inflammation in the lungs and minor pathologies in the liver and brain

**Figure 4.**
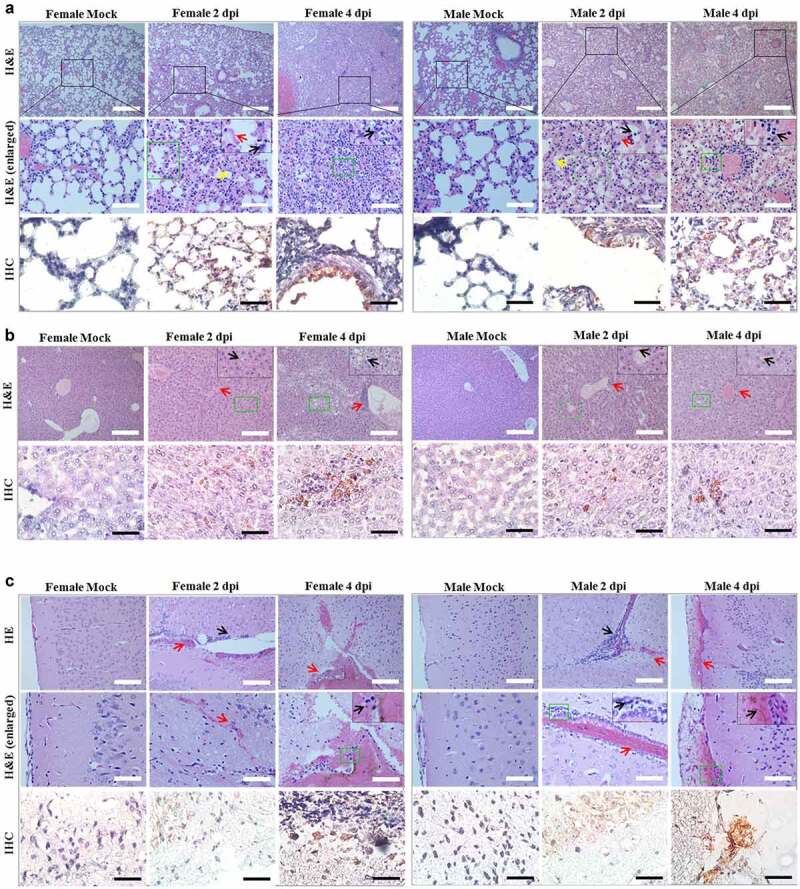
Histological examination of Roborovski SH101 hamster infected with SARS-CoV-2. In section images, green box area is enlarged again and shown at the top right black box. (a) The representative images of the H&E and IHC of the lungs of 2-month-old *P. roborovski* SH101 at 2 and 4 dpi of SARS-CoV-2. Multifocal interstitial pneumonia with thickened alveolar septa (yellow arrows) and infiltration of fibrin and mononuclear cells (black arrows) are indicated. Hyaline membrane is also observed at 2dpi (red arrows). SARS-CoV-2 antigen expression is colocalized with areas of peribronchial and alveolar epithelial cells as shown in IHC for SARS-CoV-2-nucleocapsid (400 ×). (b) The representative images of the H&E and IHC of the livers of 2-month-old *P. roborovski* SH101 at 2 and 4 dpi of SARS-CoV-2 showing pathologies. Focal and intraportal lymphoid cell aggregation and multifocal fatty changes are indicated by red and black arrows, respectively. SARS-CoV-2 antigen expression is colocalized in few liver cells as shown in IHC for SARS-CoV-2-nucleocapsid (100 ×). (c) The representative images of the H&E and IHC of the brains of 2-month-old *P. roborovski* SH101 showing pathologies at 2 and 4 dpi. Subarachnoid hemorrhage and lymphocyte focal infiltration are indicated by red and black arrows, respectively. SARS-CoV-2 antigen expression is colocalized in few subarachnoid as shown in IHC sections for SARS-CoV-2-nucleocapsid (100 ×). The scale bars represent 100 μm for 100 × and 20 μm for 400 ×

After observing that Roborovski hamster SH101 infected with SARS-CoV-2 closely represented the clinical manifestations of COVID-19 in humans, we examined the histopathological changes in each organ of the hamsters (Figure 4). Slides were carefully evaluated by a pathologist blinded to the treatment groups. As shown in Figure 4(a), pathological examinations revealed severe inflammatory lesions in the lungs of the infected SH101 hamsters starting from 2 dpi. The lesions in the lungs were the typical features of severe viral pneumonia, as shown in diffuse alveolar damage (DAD) with hyaline membrane formation at 2 dpi. The DAD pattern showed a mixture of exudative and proliferative features. Multifocal interstitial pneumonia with thickened alveolar septa and infiltration of fibrin and mononuclear cells were obvious. Immunohistochemistry also revealed the SARS-CoV-2 antigen was present in the nucleus and cytoplasm of bronchiolar and alveolar epithelial cells. The inflammatory lung lesions were extended across larger areas without any alveolar spaces due to the severity of inflammation on the last day, 4 dpi.

In addition to the pulmonary injury, slight pathological damages were also observed in the livers of the infected SH101 hamsters (Figure 4(b)). The pathological damages were observed in 4 out of the 6 livers at 2 dpi and 6 out of the 6 livers at 4 dpi, along with histological examinations. Multifocal fatty changes and portal lymphocytic infiltration observed in the livers are shown in Figure 4(b). Immunochemistry also revealed the localization of SARS-CoV-2 antigen in liver cells. The hepatic injury also strongly supported that the SH101 hamsters infected with SARS-CoV-2 closely mimicked COVID-19 in humans [40,41].

In brain specimens, like the liver, we also observed a focal infiltration of lymphocyte and/or subarachnoid hemorrhage in 4 out of the 6 brains at 2 dpi and 4 out of the 6 brains at 4 dpi (Figure 4(c)), suggesting potential neuroinflammation just like as in the case of COVID-19 disease [42,43]. Moreover, extravasation of red blood cells and hemosiderin pigments with focal infiltration of lymphocytes was also observed in the damaged brains. Immunochemistry also revealed the localization of SARS-CoV-2 antigen in subarachnoid cells. In terms of frequent neurological complications in COVID-19, the neurological damages (Figure 4c) in the infected SH101 hamsters also recapitulated the important clinical features of COVID-19. Despite high levels of SARS-CoV-2, the trachea was not damaged by SARS-CoV-2 infection, demonstrating another piece of similarity to COVID-19 [44]. The undamaged trachea despite the high viral titers implies that the trachea may function as an exhauster in the hamsters like the human counterpart. Other than these organs, no obvious histopathological changes were observed in the stomach, intestine, heart, kidney, and spleen (Figure S1).

### Comparison of roborovski SH101 with the hACE2 transgenic mouse and syrian hamster to study COVID-19

**Figure 5.**
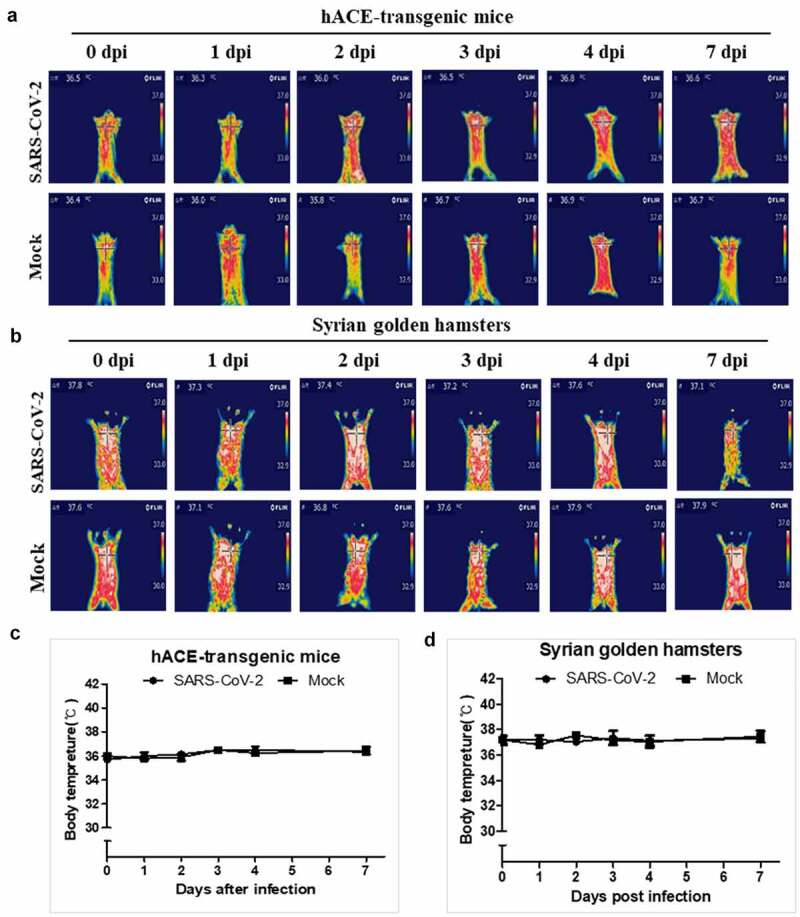
The body temperature changes of the hACE-transgenic mice and Syrian golden hamsters after infection of SARS-CoV-2. (a, b) The representative infrared thermographic images of the 2-month-old male hACE-transgenic mice (a) and 3-month-old male Syrian golden hamsters (b) at the indicated dpi of SARS-CoV-2. (c, d) The body temperatures on the chest, as close as possible to the lung, of the male hACE transgenic mice (c) and male Syrian golden hamster (d) at the indicated dpi (n = 6). The body temperatures were measured by selecting the highest temperature spot on the thermal images and are presented as mean ± SD

**Figure 6.**
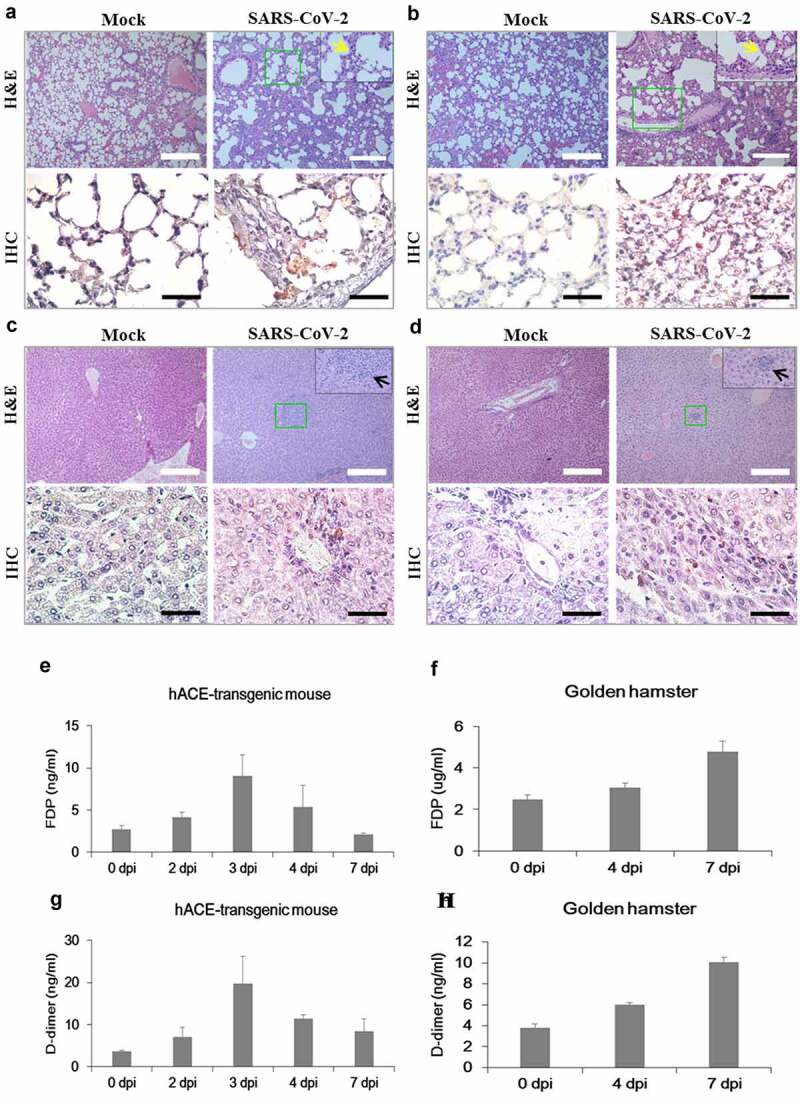
Histological examination, thrombosis, and viral replication in the hACE-transgenic mice and Syrian golden hamsters infected with SARS-CoV-2. (a-d) The representative images of the H&E and the IHC sections of the lungs (a, b) and the livers (c, d) of the 2-month-old hACE-transgenic mice (a, c) and 3-month-old Syrian golden hamsters (b, d) at 7 dpi of SARS-CoV-2 showing pathologies. Multifocal interstitial pneumonia with thickened alveolar septa (yellow arrows) and lymphoid cell aggregation (black arrows) are indicated in H&E staining. SARS-CoV-2 antigen expression is detected in the lung and liver in IHC for SARS-CoV-2-nucleocapsid (400 ×). (e, f) The levels of fibrin degradation products (FDP) in the plasma of the 2-month-old hACE-transgenic mice (e) and 3-month-old Syrian golden hamsters (f) at the indicated dpi (n = 3). (g, h) The levels of D-dimer in the plasma of the hACE-transgenic mice (E) and Syrian golden hamsters (F) after infection (n = 3). (i, j) The viral RNA levels in the primary organs, lung, brain, stomach, intestine, trachea, liver, blood, heart, kidney, and spleen, of the male hACE-transgenic mice (i) and the male Syrian golden hamsters (j) measured by RT-qPCR at the indicated dpi of SARS-CoV-2. Data are present as mean ± SD (n = 3). The scale bars represent 100 μm for 100 × magnifications

Among the various animal species investigated as an animal model for COVID-19, the hACE2 transgenic mice and Syrian hamsters are the most widely used animal models to study human COVID-19. However, despite their popularity in COVID-19 studies, infections of the hACE2 transgenic mice or Syrian hamsters with SARS-CoV-2 resulted in infection mostly in the respiratory system. Considering their broad use in COVID-19 researches, these two animal models were analyzed in comparison with the SH101 hamster after SARS-CoV-2 infection. In agreement with previous studies, our experiments using hACE2 transgenic mice and Syrian hamsters confirmed their certain degree of sensitivity to SARS-CoV-2 (Figures 5 and 6, Figure S2 and S3) but failed to observe representative symptoms of COVID-19 such as fever induction, shaking chills, and the right-over-left predominated pneumonia.

The fever induction and shaking chills are the most common and primary symptoms of COVID-19 that can be observed even in mild cases [33,34]. For the first time, we were able to show the cardinal symptoms of COVID-19, fever induction, shaking chills, and respiratory problems, in a SARS-CoV-2-infected animal just like in the human counterpart (Figure 1-4, Figure S4 ~ S7 and Video S1 ~ S4), but not in other models (Figures 5 and 6).

Both hACE2 transgenic mice and Syrian golden hamsters with SARS-CoV-2 infection demonstrated a certain degree of organ damages – heavily damaged lungs and slightly damaged livers but no detectable pathology in the brains (Figure 6(a,d) and Figure S3). In addition, the levels of fibrin degradation products, D-dimer and FDP, were increase till 3 dpi and then returned to normal level by 7 dpi in the hACE-transgenic mice (Figure 6(e,g)) while both levels increased till 7 dpi in the Syrian golden hamsters (Figure 6(f,h)). The result implies the higher susceptibility of Syrian golden hamsters compared to hACE-transgenic mice. Interestingly, the viral RNA copies in the lungs of SH101, the hACE2 transgenic mice, and Syrian hamsters were not much different – 6.4 ± 0.4 (4 dpi), 5.7 ± 0.3 (4 dpi), and 5.6 ± 0.2 (4 dpi), respectively (Figure 6(i,j)). The dramatic differences of clinical manifestations between current models and SH101 despite the similar range of virus titers (Figures 3(e,f and 6(i,j))) suggest that a physiological makeup of *P. roborovskii* SH101 would be different from those of the other two animals.

It has been well studied that young animals were significantly less sensitive to SARS-CoV-2 [45,46]. While hACE2 transgenic mice and Syrian hamsters displayed age-dependent sensitivity, SH101 hamsters as young as one-month-old were able to show serious symptoms by SARS-CoV-2 infection (Figure S4 ~ S7). The symptomatic severities of the 1-month-old hamsters assessed by the organ damages were comparable to those of 2-month-old (Figure 4 and Figure S1, S5, S6). The virus titers of the young SH101 were 7.0 ± 0.3 in the lung at 4 dpi (Figure S7) like those of adults (Figure 3(e,f)). Despite the general similarity between the young and adult hamsters, SARS-CoV-2 infection led to a 100% mortality rate in the young SH101 hamsters by 4 dpi.

## Discussion

Multiple animal species have been investigated as an animal model for COVID-19 since the onset of the pandemic [45,46]. Among the developed animal models to study COVID-19, the hACE2 transgenic mice and Syrian hamsters are the most favorable animal models currently because of the merit of easy handling of small animals [13,15]. Despite their popularity in the COVID-19 studies, the widespread use of these two animals for SARS-CoV-2 infection needs to be reconsidered due to their limits in the implementation of human COVID-19. This work confirmed that SARS-CoV-2 infections are mostly localized in the respiratory systems of the hACE2 transgenic mice and Syrian hamsters (Figures 5, 6 and Figure S2, S3) in agreement with previous studies [13,15]. COVID-19, however, is known to display a various degree of systemic infection in addition to localized respiratory infection, even in mild cases, indicating that the hACE2 transgenic mice and Syrian hamsters are not ideal models to study human COVID-19.

Recently, Trimpert et al. reported that another strain (Hong Kong strain) of Roborovski dwarf hamster (*P. roborovskii*) showed a rapid and fatal course of SARS-CoV-2 infection [47]. The Hong Kong strain caused severe acute diffuse alveolar damage and hyaline microthrombi in the lungs after SARS-CoV-2 infection, which were frequently described in COVID-19 patients. Although the hamster strain was highly sensitive to SARS-CoV-2, the strain did not show a systemic infection as in the case of this work. It seems that the genetic background of the Hong Kong strain differs from our strain of Roborovski dwarf hamster. A future comparative genetic analysis would provide an important piece of information on how SARS-CoV-2 leads to systemic infection in human.

The repertory of clinical presentations of COVID-19 is far exceeding respiratory symptoms, including diarrhea, loss of sense of smell or taste, neuroinflammation manifested as encephalitis, meningitis, acute cerebrovascular disease, GBS, multisystem inflammatory syndrome, thrombosis, and hyperfibrinolysis [4,5]. Given the nature of the systemic infection of COVID-19, an ideal COVID-19 animal model should represent not only most respiratory infections but also other systemic infections observed in human COVID-19. Unfortunately, none of the current COVID-19 animal models reproduce the salient clinical and pathological features of human COVID-19 [22]. Even primate models, the closest animal models to humans, did not reproduce the clinical symptoms from the systemic infection of human COVID-19 [16,45]. It is therefore pivotal to develop an animal model for the systemic infection replicating most clinical aspects of human COVID-19. In this regard, *P. roborovskii* SH101 would be an ideal animal model for evaluating antiviral therapeutic agents and vaccines, as well as understanding the pathogenesis of human COVID-19. Interestingly, *P. roborovskii* SH101 infected with SARS-CoV-2 recapitulated the very interesting COVID-19-specific facet, right-predominated pneumonia (Figure 1(e)), which is uniquely observed in human COVID-19.

Roborovski hamster strain SH101 in this study not only reproduced the salient clinical and pathological features of human COVID-19 but also demonstrated unusual rapid progression once infected. The SARS-CoV-2-infected hamsters were observed with fever induction early on 1 ~ 2 dpi, followed by rapid progression into the terminal stage to death by 3 ~ 4 dpi (Figure 2). It should be noted that this acute symptom might require careful design and attention during the execution of experiment. Taken together, its unusually rapid progression in addition to clinical presentations would provide an ideal animal model to investigate disease mechanisms and to develop drugs and vaccines for COVID-19. These characteristics of the Roborovski hamster SH101 model seem to provide an animal model that will fill a different niche than primate models. It is also very important to test its utility as animal model for the infection for SARS-CoV-2 variants in further studies.

SARS-CoV-2 is an RNA virus that quickly evolves into various strains by rapid mutations. In fact, multiple strains of SARS-CoV-2 have already emerged [48], and thus the development of animal models recapitulating most of the clinical manifestations of COVID-19 would be of utmost importance not only for overcoming COVID-19 but also for understanding the pathogenesis. Further genome analyses of Roborovski hamster strain SH101 in comparison with SARS-CoV-2-resistant hamsters or with SARS-CoV-2-susceptible hamsters for the local respiratory infection, such as Syrian hamsters, would provide critical clues for the understanding of human COVID-19.

## Supplementary Material

Supplemental Material

## Data Availability

The data that supplementary video the findings of this study are openly available in “figshare” at https://figshare.com/s/448f2198b5db48402fd0, https://figshare.com/s/3bb52d39a33c1b3c72d2, https://figshare.com/s/0c1b7ba6329a6f9fc1df and https://figshare.com/s/40689f34114dc42f4dba.
