# Supplementary material for: Roborovski hamster (Phodopus roborovskii) strain SH101 as a systemic infection model of SARS-CoV-2
Source: Virulence. 2021 Sep 14;12(1):2430–42. doi: 10.1080/21505594.2021.1972201 (PMC8451461; doi:10.1080/21505594.2021.1972201)
Supplement: Supplemental Material [file KVIR_A_1972201_SM8361.docx]

**Supplementary Data**

**Supplementary Table 1.** The summary of clinical parameters and histology of SARS-CoV-2 infections in Roborovski hamster strain SH101, hACE2 transgenic mice, and Syrian golden hamster models.

|  | **Roborovski SH101**  **-young** | **Roborovski SH101** | **hACE2 Tg mice** | **Syrian golden hamster** |
| --- | --- | --- | --- | --- |
| **Viral load in lung** | 10e7.0 | 10e6.4 (female),  10e5.9 (male) | 10e5.1 | 10e5.4 |
| **Viral load in trachea** | 10e6.3 | 10e6.3 (female),  10e6.4 (male) | 10e6.0 | 10e4.8 |
| **Viral detection in non-respiratory organs by IHC** | brain, liver, intestine | brain, liver, intestine | brain, liver | brain, liver |
| **Viral detection in non-respiratory organs by RT-qPCR** | brain, liver, intestine, heart, kidney, spleen | brain, liver, intestine, heart, kidney, spleen | brain, liver, intestine, heart, kidney, spleen | brain, liver, intestine, heart, kidney, spleen |
| **Body weight reduction** | 2 dpi | 2 dpi | 7 dpi | 3 dpi |
| **Body temperature reduction** | 2 dpi | 2 dpi | no | no |
| **Fever & shaking chills** | Fever (1 dpi)  shaking chills (2 dpi) | Fever (1 dpi)  shaking chills (3 dpi) | no | no |
| **Affected lung** | 75% | 71% (female),  67% (male) | 44% | 31% |
| **Hyaline membranes** | Yes | Yes | Yes,  small | Yes,  small |
| **Alveolar oedema** | Yes | Yes | Yes,  small | Yes,  small |
| **Leukocyte infiltration** | Yes | Yes | Yes,  small | Yes,  small |
| **Survival** | 0% | 17% | 67% | 100% |

**Figure S1 ~ S4 containing**


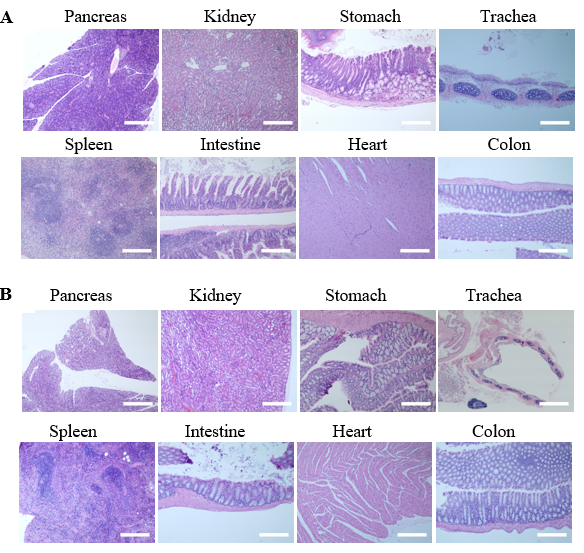


**Supplementary Figure 1.** The histological images of the primary organs of *P.* *roborovski* SH101 infected with SARS-CoV-2. The representative images of the H&E-stained histological and IHC sections without any pathologies in the primary organs of female (A) and male (B) *P. roborovski* SH101 at 4 dpi of SARS-CoV-2. The scale bars represent 100 μm for 100 × magnifications.

**
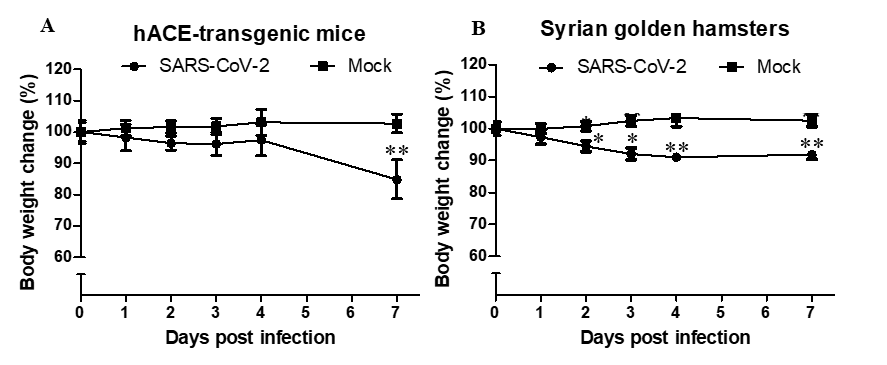
**

**Supplementary Figure 2.** The body weight changes for the hACE-transgenic mice and Syrian golden hamsters infected with SARS-CoV-2. The body weights of the male hACE-transgenic mice (A**)** and the male Syrian golden hamsters (B) infected with SARS-CoV-2 were measured daily for 7 days (n = 6). Data are presented as mean ± SD. The statistical significances are marked on the graphs as * *P* < 0.05 and ** *P* < 0.01.


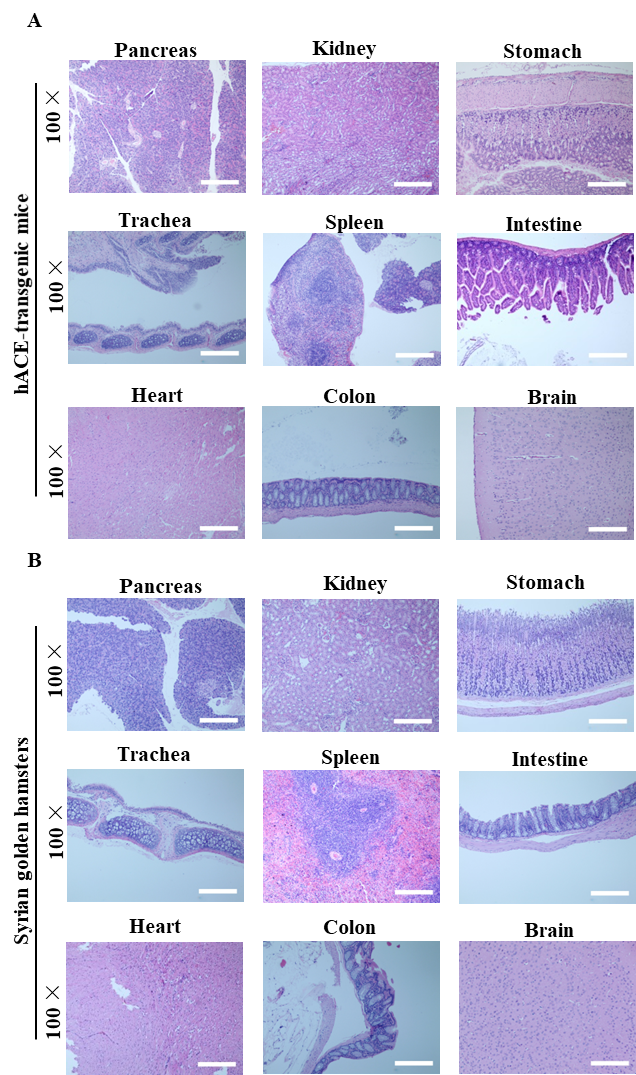


**Supplementary Figure 3.** The histological examination results of the primary organs showing no pathological damages of the hACE-transgenic mice and Syrian golden hamsters infected with SARS-CoV-2. (A, B) The representative images of the H&E-stained histological sections of the primary organs of the 2-month-old male hACE-transgenic mice (A) and 3-month-old male Syrian golden hamsters (B) at 7 dpi of SARS-CoV-2. The scale bars represent 100 μm for 100 × magnification.


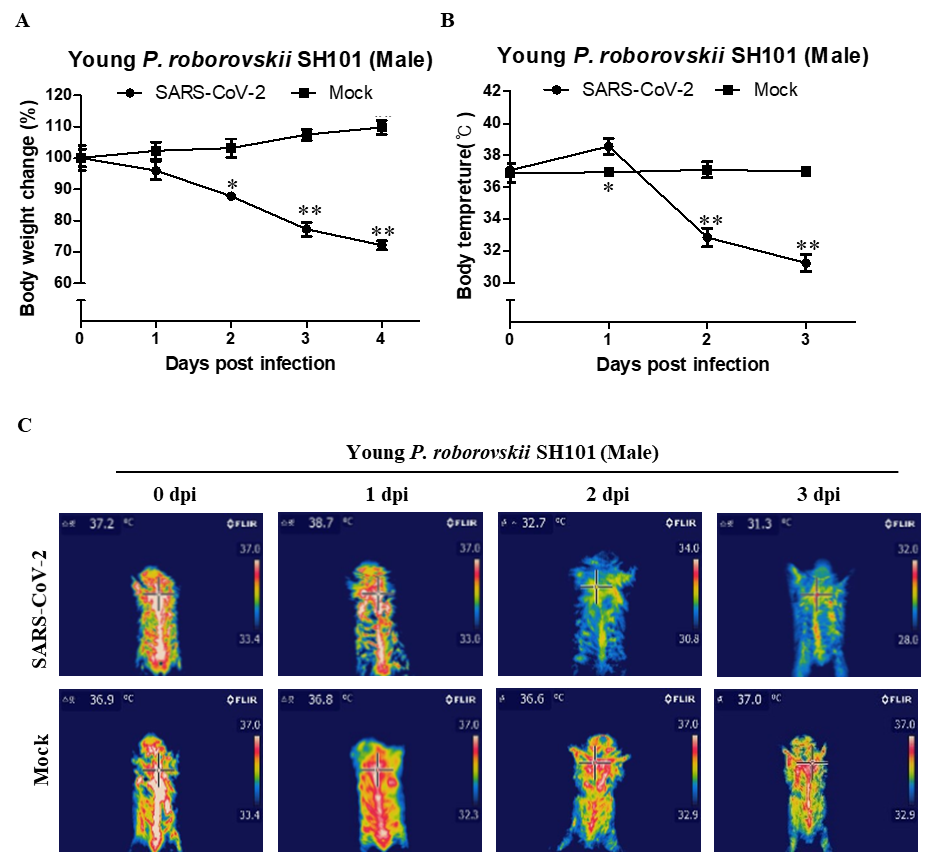


**Supplementary Figure 4.** The changes of the body weight and the body temperatures of young *P. roborovski* SH101 post-infection of SARS-CoV-2. (A) The body weights of the 1-month-old male *P. roborovski* SH101 post-infection of SARS-CoV-2 were measured daily for 4 days (n=3). (B) The surface body temperatures of the 1-month-old male *P. roborovski* SH101 post-infection of SARS-CoV-2 were measured daily for 4 days (n=3). (C) The representative infrared thermographic images of the 1-month-old male *P. roborovski* SH101 post-infection of SARS-CoV-2. The surface body temperatures were measured by selecting the highest temperature spot on the thermal images where the lung is located, and are presented as mean ± SD. The statistical significances are marked on the graphs as * *P* < 0.05 and ** *P* < 0.01.

**
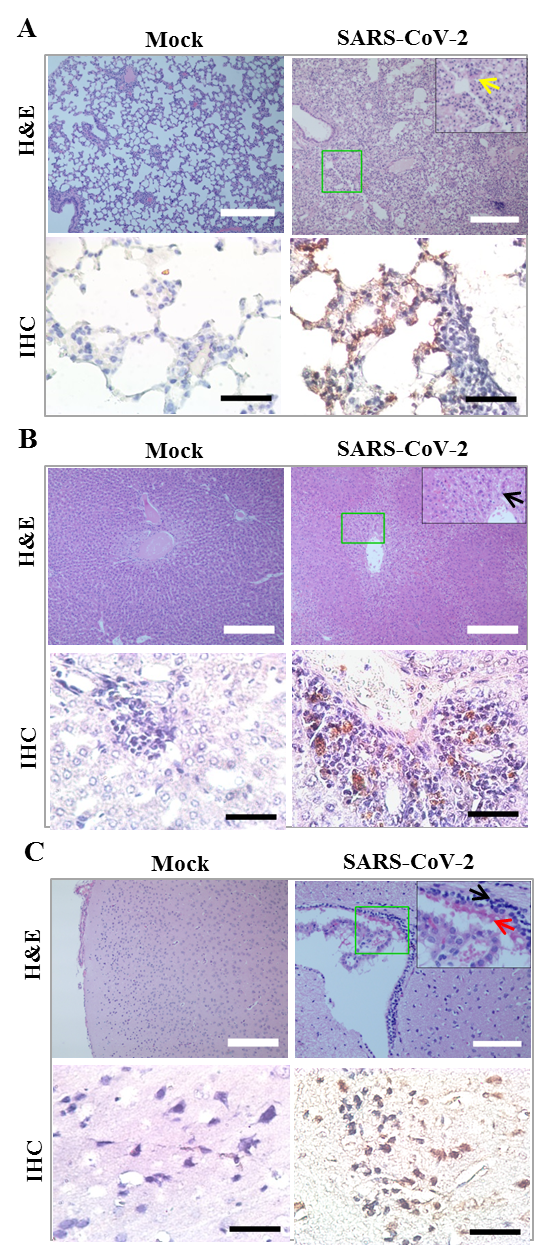
**

**Supplementary Figure 5.** The results of histological examination of the primary organs showing pathologies of young *P. roborovski* SH101 post-infection of SARS-CoV-2. (A-C) The representative images of the H&E-stained histological and IHC sections of the lungs (A), livers (B), and brains (C) of the 1-month-old *P. roborovski* SH101 at 4 dpi. In H&E staining, green box area is enlarged and shown at the black box. Multifocal interstitial pneumonia with thickened alveolar septa (yellow arrows) is indicated in the lung. Lymphoid cell aggregation is indicated by black arrows in the liver and brain. Subarachnoid hemorrhage is indicated by red arrows in the brain. SARS-CoV-2 antigen expression is detected in the lung, liver, and brain with IHC for SARS-CoV-2-nucleocapsid (400 ×). (B)The scale bars represent 100 μm for 100 × magnification.

**
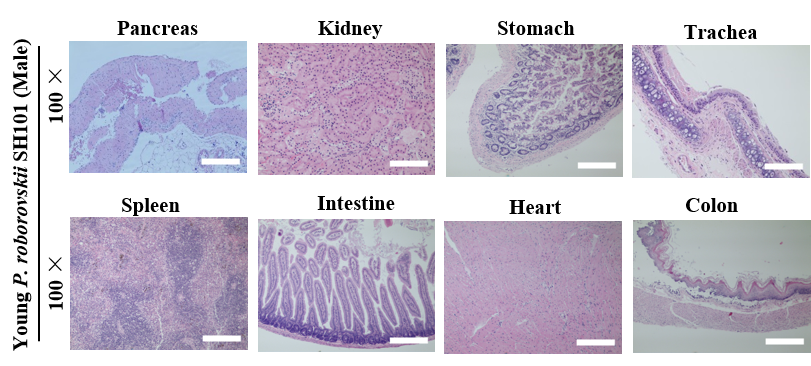
**

**Supplementary Figure 6.** The histological examination results of the primary organs showing no pathological damages of young *P. roborovski* SH101 infected with SARS-CoV-2. The representative images of the H&E-stained histological sections of the undamaged primary organs of the 1-month-old *P. roborovski* SH101 infected with SARS-CoV-2 at 4 dpi are shown. The scale bars represent 100 μm for 100 × magnification.


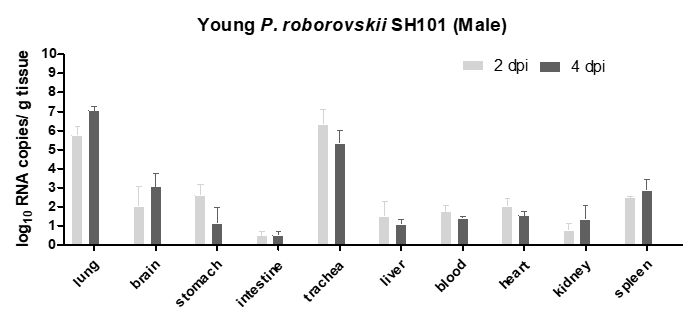


**Supplementary Figure 7.** SARS-CoV-2 replication in young *P. roborovski* SH101 after SARS-CoV-2 infection. The viral RNA levels in the lung, brain, stomach, intestine, trachea, liver, blood, heart, kidney, and spleen of the 1-month-old *P. roborovski* SH101 were measured by RT-qPCR 2 and 4 dpi of SARS-CoV-2 (n = 3). Data are present as mean ± SD.

**Video S1 ~ S4 containing**

**Video S1:** General behavioral symptoms of male *P. roborovski* SH101 infected with SARS-CoV-2. (https://figshare.com/s/448f2198b5db48402fd0)

**Video S2:** The sneezing symptom of *P. roborovski* SH101 infected with SARS-CoV-2.

(https://figshare.com/s/3bb52d39a33c1b3c72d2)

**Video S3:** The shaking chills of *P. roborovski* SH101 infected with SARS-CoV-2. (https://figshare.com/s/0c1b7ba6329a6f9fc1df)

**Video S4:** General behavioral symptoms of young male *P. roborovski* SH101 infected with SARS-CoV-2. (https://figshare.com/s/40689f34114dc42f4dba)
